# LncRNA TCONS_00021861 is functionally associated with drought tolerance in rice (*Oryza sativa* L.) via competing endogenous RNA regulation

**DOI:** 10.1186/s12870-021-03195-z

**Published:** 2021-09-07

**Authors:** Jiajia Chen, Yuqing Zhong, Xin Qi

**Affiliations:** School of Chemistry and Life Science, Suzhou University of Science and Technology, No.1 Kerui Road, 215011 Suzhou, China

**Keywords:** Drought stress, lncRNA, Network, ceRNA, IAA

## Abstract

**Background:**

Water deficit is an abiotic stress that retards plant growth and destabilizes crop production. Long non coding RNAs (lncRNAs) are a class of non-coding endogenous RNAs that participate in diverse cellular processes and stress responses in plants. lncRNAs could function as competing endogenous RNAs (ceRNA) and represent a novel layer of gene regulation. However, the regulatory mechanism of lncRNAs as ceRNA in drought stress response is yet unclear.

**Results:**

In this study, we performed transcriptome-wide identification of drought-responsive lncRNAs in rice. Thereafter, we constructed a lncRNA-mediated ceRNA network by analyzing competing relationships between mRNAs and lncRNAs based on ceRNA hypothesis. A drought responsive ceRNA network with 40 lncRNAs, 23 miRNAs and 103 mRNAs was obtained. Network analysis revealed TCONS_00021861/miR528-3p/YUCCA7 regulatory axis as a hub involved in drought response. The miRNA-target expression and interaction were validated by RT-qPCR and RLM-5’RACE. TCONS_00021861 showed significant positive correlation (*r* = 0.7102) with YUCCA7 and negative correlation with miR528-3p (*r *= -0.7483). Overexpression of TCONS_00021861 attenuated the repression of miR528-3p on YUCCA7, leading to increased IAA (Indole-3-acetic acid) content and auxin overproduction phenotypes.

**Conclusions:**

TCONS_00021861 could regulate YUCCA7 by sponging miR528-3p, which in turn activates IAA biosynthetic pathway and confer resistance to drought stress. Our findings provide a new perspective of the regulatory roles of lncRNAs as ceRNAs in drought resistance of rice.

**Supplementary Information:**

The online version contains supplementary material available at 10.1186/s12870-021-03195-z.

## Background

Rice (*Oryza sativa* L.) is a major staple crop worldwide and is highly susceptible to water deficit. Drought stress is a limiting factor in rice production and has caused severe losses on the rice yields over the past decades. Plants can adapt to stresses by modulating multiple signaling pathways, among which noncoding RNAs play essential roles [1].

Long non-coding RNAs (lncRNAs) are a diverse class of ncRNAs larger than 200 bp in length without apparent coding potential. LncRNAs play widespread roles in virtually every biological process in plants [2]. RNA sequencing technologies have provided powerful tools for transcriptomic research and shed light into the lncRNAs of both model and crop plants, e.g. Arabidopsis [3, 4], wheat [5], maize [6, 7], rice [8, 9] and *Medicago truncatula* [10]. In particular, many stress-responsive lncRNAs were revealed in rice. lncRNAs were found to mediate anti-pathogen immunity [1, 7] as well as the response to abiotic stresses such as drought [11], salinity [12], heavy metal [13, 14] and nutrient deficiency [15].

Competing endogenous RNA (ceRNA) is a widespread type of gene regulation for lncRNA. LncRNAs acting as potential ceRNAs harbor microRNA response elements (MREs), thereby they compete with mRNAs for shared miRNA and thus regulate miRNA-mediated gene silencing. The first ceRNA was reported in Arabidopsis and the study identified an endogenous lncRNA, IPS1 (Induced by Phosphate Starvation 1). IPS1 acts as target mimic of miR399 and inhibits miR399-induced cleavage of PHO2, which modulates phosphate accumulation [16].

Many lncRNA acting as endogenous miRNA target mimics (eTMs) have been identified in plants. lnc_253 and lnc_973 may function as eTMs for miR156e under salt stress in cotton [17]. In tomato, lncRNA23468 acts as a ceRNA to modulate NBS-LRR genes by decoying miR482b under *Phytophthora infestans* [18]. Feng et al. profiled lncRNAs in Cd-stressed rapeseed and identified 67 lncRNAs as competing target mimics for 36 Cd responsive miRNAs [19]. In pigeonpea, lncRNA_1231 during flowering sequesters miR156b through the target mimicry manner leading to increased expression of flower-specific SPL-12 transcription factor and regulates flower development [20]. lncRNA39026 acts as eTMs of miR168a and may decoy miRNAs to upreglate SlAGO1, and enhances tolerance to Phytophthora infestans [21]. In Cassava, linRNA159 and linRNA340 were predicted to be target mimics of miR164 and miR169 under cold stress [22]. In winter wheat, lncRNAs were found to competitively bind with miR398 and regulate CSD1 expression, which improves cold resistance [5]. However, only a little proportion of these lncRNAs have been experimentally and functionally characterized.

In the current study, we systematically analyzed the transcriptional landscape of drought-responsive lncRNAs in rice to identify differentially expressed lncRNAs, miRNAs and mRNAs. A deregulated lncRNA-associated ceRNA network was constructed according to the ceRNA hypothesis. A ceRNA regulatory triplet were identified, validated and functionally characterized. Finally, a hypothetic model based on lncRNA-miRNA-mRNA-IAA signaling was established to reveal the role of lncRNAs in drought response. Our findings may provide insights into the mechanism of lncRNAs in drought response regulation.

## Results

### Transcriptome sequencing of rice in response to drought stress

**Fig. 1:**
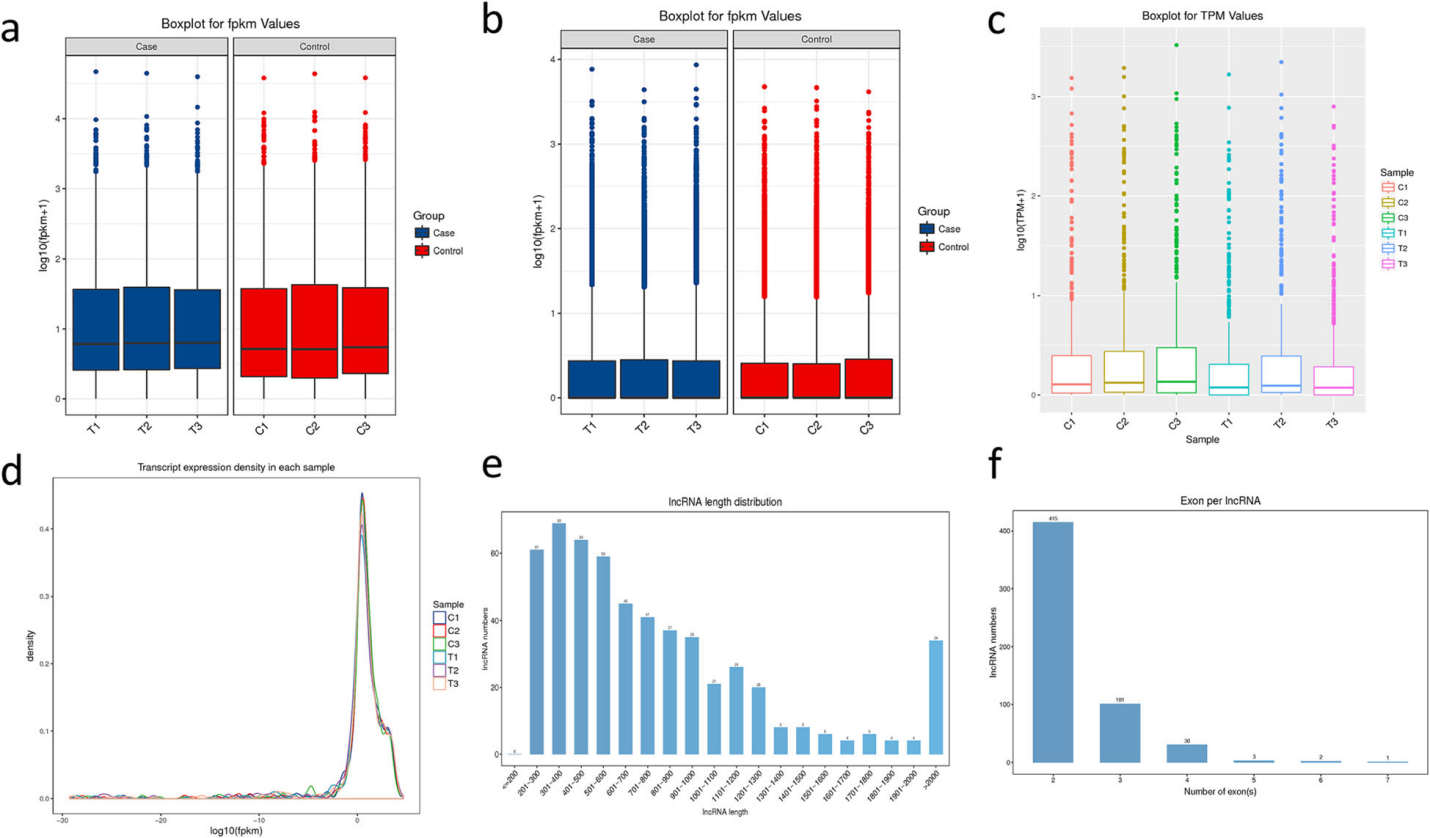
Characteristics of lncRNAs expression. **a**: Expression levels of lncRNAs. **b**: Expression levels of mRNAs. **c**: Expression levels of miRNAs. **d**: Expression density of lncRNAs. **e**: Lengths distributions of lncRNAs. **f**: Exon numbers distribution of lncRNAs

High-throughput paired-end sequencing (Illumina) was performed in control and PEG treated rice seedlings. A total of 578.64 million raw reads were obtained from 6 samples. After adapter trimming and filtration, 564.11 million clean reads were obtained and 93.79 % were mapped to the rice reference sequence. A total of 301,926 transcripts were reconstructed with cufflink 2.0. We obtained 542 high-confidence putative lncRNAs, including 48 intronic lncRNAs, 331 antisense lncRNAs and 163 intergenic lncRNAs. Most of lncRNAs lie between FPKM value 1–10. Box plots (Fig. 1a and c) illustrate the overall distribution of the expression level of lncRNA, mRNA and miRNA respectively. The lncRNA density distribution was given in Fig. 1d. As shown in Fig. 1e, most lncRNAs(93.81 %)were 201–1900 bp in length and 93.47 % of lncRNAs had no more than 3 exons (Fig. 1f). The short transcript length and low exon number were two common features of lncRNAs.

### Expression Profiles of lncRNAs, miRNAs and mRNAs

**Fig. 2:**
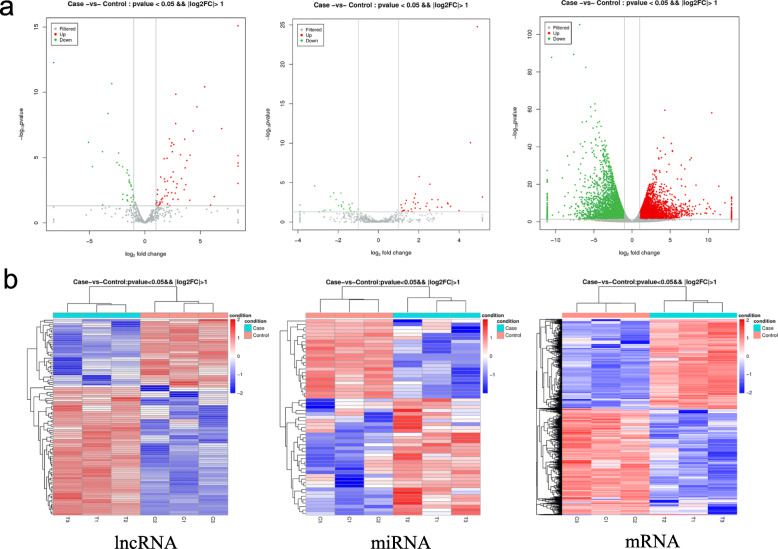
Analysis of differentially expressed of lncRNAs, miRNAs and mRNAs. **a**: volcano plot of the differential lncRNAs, miRNAs and mRNAs. Red and green dots indicate the upregulation and downregulation, respectively. Grey dots represent non-differential expression. **b**: Heatmap for the differential lncRNAs, miRNAs and mRNAs. The red indicates upregulation, whereas blue represents downregulation

Deferential expression was analyzed with the cutoff log_2_FC > 1 and adjusted *P*-value < 0.05. DE lncRNAs, miRNAs and mRNAs were displayed in volcano plot (Fig. 2a) and heatmap (Fig. 2b). A total of 98 DE-lncRNAs (64 upregulated and 34 downregulated), 57 DE-miRNAs (34 upregulated and 23 downregulated) and 7020 DE-mRNAs (3222 upregulated and 3798 downregulated) were identified in the PEG treated group as against the control, respectively.

### ceRNA Network was constructed in rice leaves

To evaluate the lncRNA-associated ceRNA interaction landscape involved in drought stressed rice, we constructed the lncRNA-mRNA-miRNA network according to the “ceRNA hypothesis” as follows:

First, miRNA-target interaction was predicted via nucleotide sequence and coexpression values, respectively. In total, we obtained 4739 miRNA-mRNA and 381 miRNA-lncRNA interactions. The lncRNA‐mRNA interaction was identified based on the Pearson correlation coefficient of the expression value.

Next, ceRNAs were filtered using ceRNA score, which was defined as the number of MREs between shared mRNAs and lncRNAs versus the total number of lncRNA MREs. For a given lncRNA-miRNA-mRNA triplet, both mRNAs and lncRNAs are targeted and negatively co-expressed with a common miRNA. As a result, 2727 lncRNA-miRNA-mRNA triplets were identified, consisting of 72 lncRNAs, 44 miRNAs and 257 mRNAs.

**Fig. 3:**
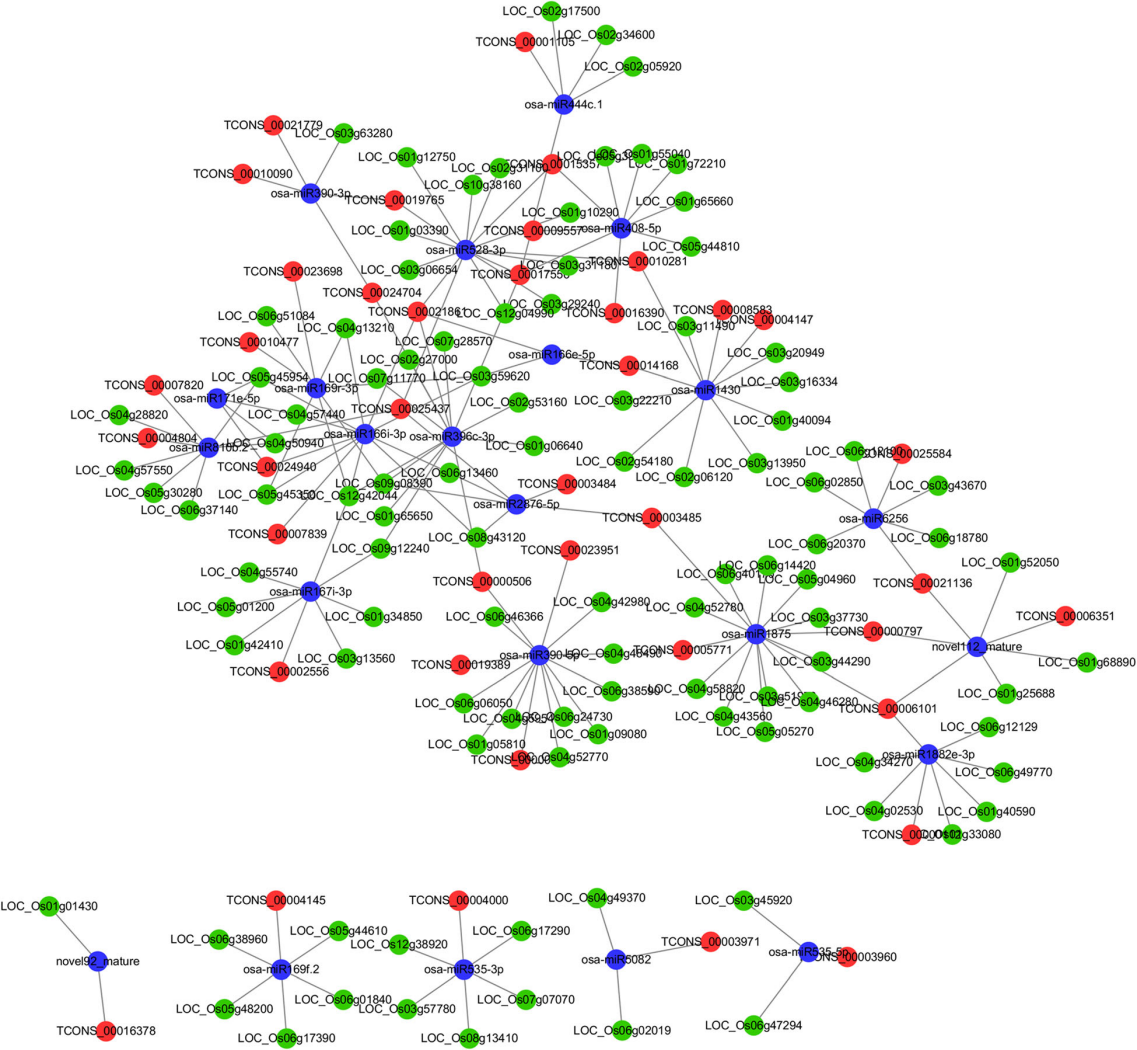
lncRNA-based ceRNA network. Red, blue and green nodes represent lncRNAs, miRNAs and mRNAs, respectively. The edges represented the competing interactions among them

Finally, to make the networks more concise and robust, we further filtered the 103 key DE mRNAs in drought response through literature mining and functional annotation. Based on the selected mRNAs implicated in drought response, we constructed a network of 180 lncRNA-miRNA-mRNA triplets, including 40 lncRNAs, 23 miRNAs and 103 mRNAs (Fig. 3).

Hub nodes with high connectivity are important to a network. In our analysis, the hub was defined as the node with highest degree. miR528-3p with the highest degree of 15 was found to be the core component of the network. Among the miR528-3p involved mRNA-lncRNA coexpression, TCONS_00021861 relative to YUCCA7 (LOC4331709, Indole-3-pyruvate monooxygenase) had most significant positive correlation (*r* = 0.89, *p* = 0.016). Therefore, the TCONS_00021861/miR528-3p/YUCCA7 triplet was chosen for further functional investigation.

### RT-qPCR confirmed the differential expression of drought-responsive lncRNAs

**Fig. 4:**
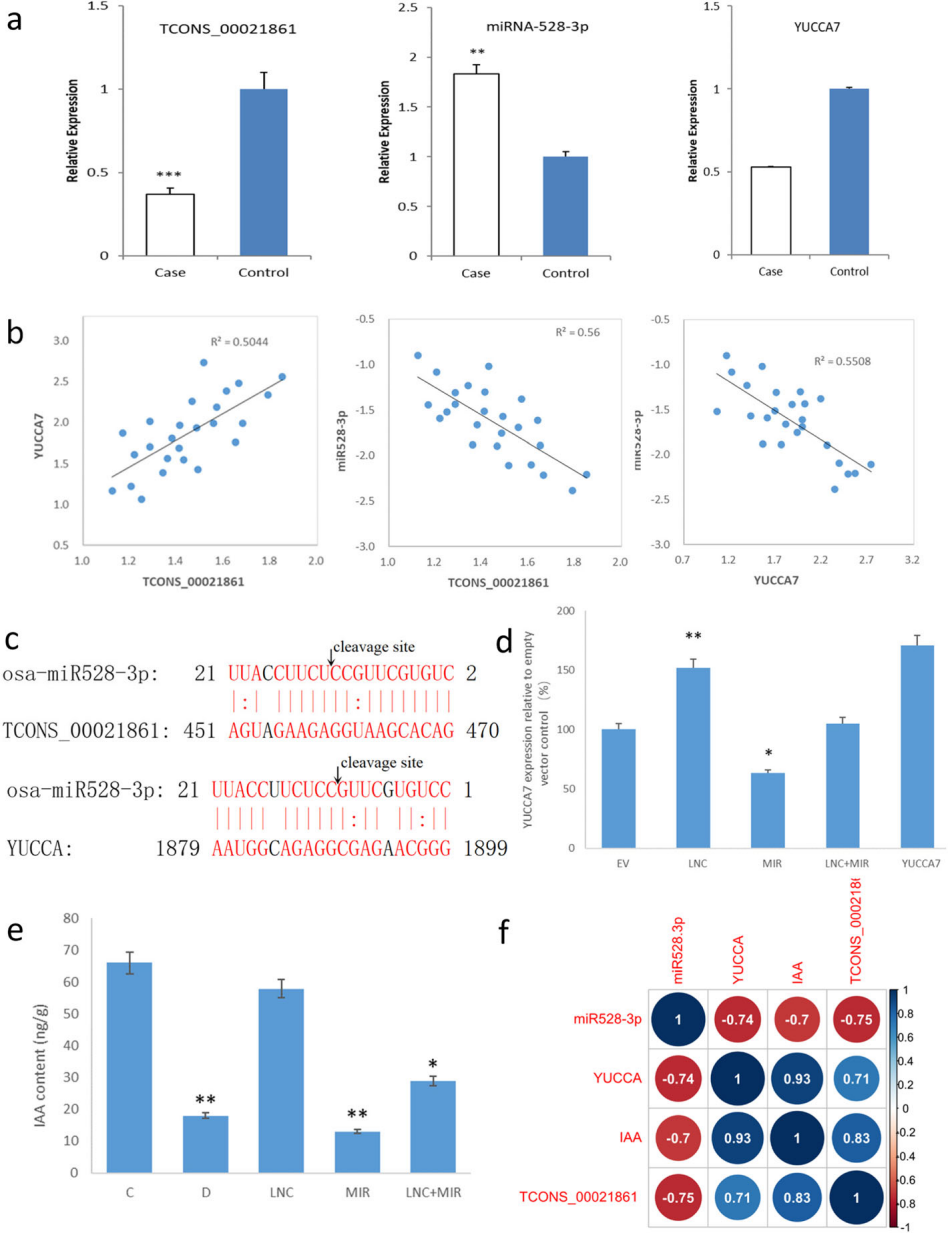
Verification of competitive interaction between TCONS_00021861/miR528-3p/YUCCA7. **a**: The expression levels of TCONS_00021861, YUCCA7 and miR528-3p were examined by RT-qPCR in drought stressed vs. control. **b**: Expression correlation between TCONS_00021861/miR528-3p/YUCCA7 in stressed leaf tissues. **c**: Potential miR528-3p cleavage sites on TCONS_00021861 and YUCCA7 identified by RLM-5′ RACE. **d**: YUCCA7 expression in response to overexpression of TCONS_00021861 and miR528-3p. **e**: IAA content of mesophyll cells determined by HPLC. **f**: Pearson correlation coefficients between TCONS_00021861/miR528-3p/YUCCA7. EV: empty vector; LNC: TCONS_00021861 overexpression; MIR: miR528-3p overexpression; LNC + MIR: co-overexpression of TCONS_00021861 and miR528-3p; C: the control without any treatment; D: treatment by 20 % PEG 6000; *P < 0.05, **P < 0.01, ***P < 0.001

RT-qPCR was performed to validate TCONS_00021861, miR528-3p and YUCCA7 expression levels. TCONS_00021861 and YUCCA7 were downregulated, whereas miR528-3p was upregulated in the samples obtained from PEG‐treated rice compared with the control samples (Fig. 4a). The results were consistent with lncRNA sequencing. We then assessed the correlations among TCONS_00021861, miR528-3p and YUCCA7. As illustrated in Fig. 4b, the significant positive correlation between TCONS_00021861 and YUCCA7, and their negative correlation to miR528-3p expression levels support the ceRNA hypothesis.

### RLM-5’ RACE confirmed cleavage of TCONS_00021861 and YUCCA7 by miR528-3p

Interaction between miR528-3p and its targets, TCONS_00021861 and YUCCA7, were experimentally confirmed by RLM-5’ RACE. After sequencing the RLM-5’ RACE PCR products, it’s found that both TCONS_00021861 and YUCCA7 had specific cleavage sites in the complementary miR528-3p sequence (Fig. ​4c).

### TCONS_00021861 specifically sequesters miR528-3p to upregulate YUCCA7

To test whether TCONS_00021861 could regulate YUCCA7 by decoying miR528-3p, we overexpressed TCONS_00021861/miR528-3p and both to obtain three independent transgenic lines. qPCR was performed to evaluate the transfection effect. As shown in Fig. 4d, overexpressed TCONS_00021861 induced the YUCCA7 levels. In contrast, the overexpression of miR528-3p reduced YUCCA7 level, and the reduction could be rescued by overexpressing TCONS_00021861 simultaneously. In summary, the data demonstrated that TCONS_00021861 is a positive regulator of YUCCA7 by sequestering miR528-3p.

### Interaction between TCONS_00021861 and miR528-3p regulated IAA pathway

Since YUCCA7 is known to regulate IAA synthesis, we inferred that TCONS_00021861 could participate in IAA biosynthesis pathway via ceRNA. To test this hypothesis, transgenic lines overexpressing TCONS_00021861/miR528-3p and both were used for the IAA content analysis. IAA content was detected by HPLC. As shown in Fig. 4e, IAA level significantly increased in transgenic plants overexpressing TCONS_00021861 whereas decreased obviously in plants overexpressing miR528-3p. However, no obvious IAA change was observed in the transgenic rice overexpressing both TCONS_00021861 and miR528-3p.

Correlation analysis between TCONS_00021861/miR528-3p/YUCCA7 found that IAA content was positively correlated with TCONS_00021861 (*r* = 0.83), YUCCA7 (*r* = 0.93) and negatively correlated with miR528-3p (*r* = -0.70) (Fig. 4f). Therefore, it’s assumed that TCONS_00021861 may function under drought treatment to upregulate the YUCCA7 concentration, which further increased the IAA level.

### Overexpression of TCONS_00021861 alleviated drought-induced growth reduction and ROS accumulation

**Fig. 5:**
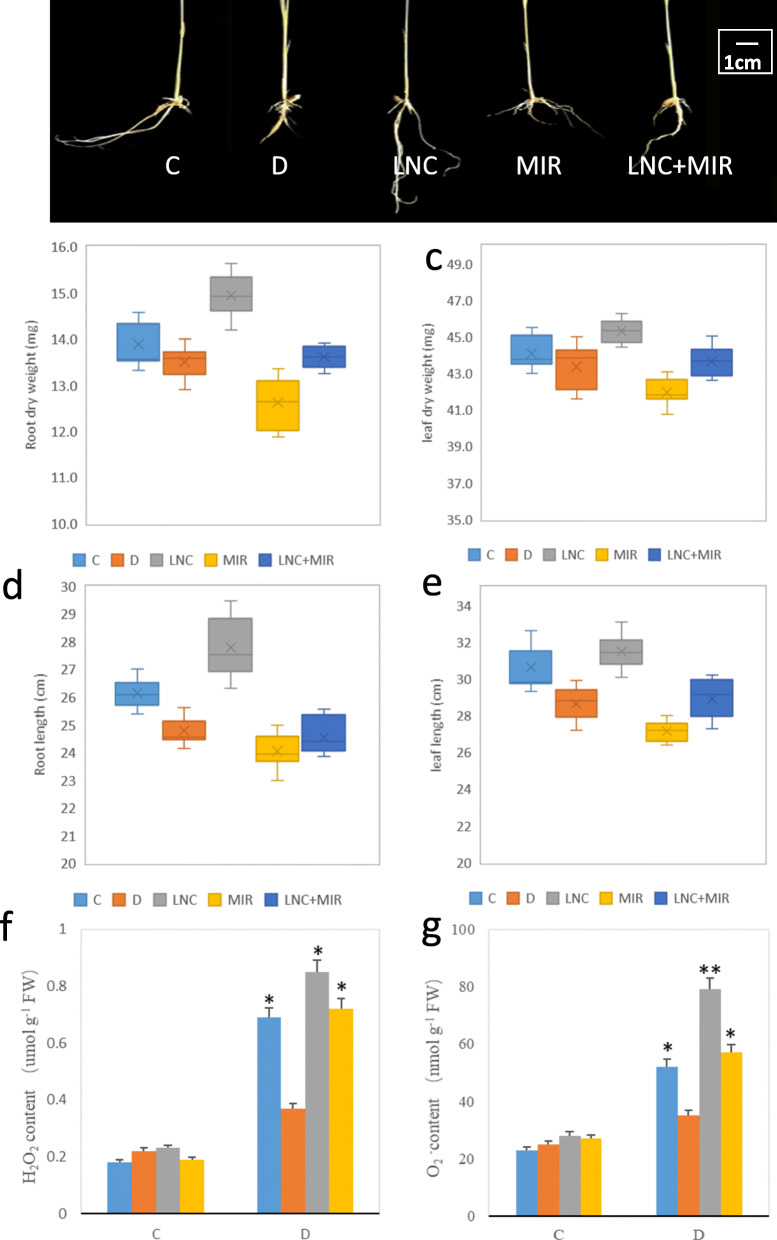
Effects of overexpressed TCONS_00021861 and miR528-3p on growth indices and ROS accumulation of drought-stressed rice seedlings. **a**: Seedlings were photographed and measured 1 week after PEG simulated drought stress. **b**: root dry weight. **c**: leaf dry weight. **d**: root length. **e**: leaf length. **f**: H_2_O_2_ content. **g**: O_2_•^−^ content. All the data represent the mean ± standard deviation (SD) of 20 rice seedlings

Plant growth indices were measured to investigate the impact of TCONS_00021861 and miR528-3p on the resistance of rice to drought stress. Transgenic lines overexpressing TCONS_00021861/miR528-3p and both were used for the growth observation. As shown in Fig. 5a-e, plant growth was affected by drought stress in terms of leaf and root length. Compared with WT, the overexpression of TCONS_00021861 increased root dry weight, leaf dry weight, root length and leaf length by 10.37 %, 4.38 %, 12.19 and 9.88 % respectively. Indices in the miR528-3p overexpression groups invariably decreased (from 7.85 to 18.25 %). However, upon co-overexpression of TCONS_00021861 and miR528-3p, growth indices were rescued (from 4.05 to 18.76 %).

Two main ROS species, H_2_O_2_ and O_2_•^−^, were quantitatively determined. As shown in Fig. 5f, before PEG treatment, H_2_O_2_ and O_2_•^−^ accumulations were relatively low and no obvious difference was observed between wildtype and transgenic lines. However, drought stress significantly increased the accumulation of H_2_O_2_, especially in miR528-3p overexpression lines. Co-overexpression of TCONS_00021861 and miR528-3p had a smaller increase range in H_2_O_2_. In TCONS_00021861 overexpression line, no obvious increase in H_2_O_2_ accumulation was detected. Similar result was observed in O_2_•^−^ accumulation (Fig. 5g). The results showed that TCONS_00021861 overexpression alleviates the accumulation of ROS species.

Interestingly, enhanced IAA content in TCONS_00021861 overexpression plants is consistent with the enhanced growth index and reduced ROS content (Fig. 4e), indicating that TCONS_00021861 could relieve drought-induced growth retardation and ROS accumulation via IAA signaling.

### Ultrastructure by Transmission Electron Microscopy (TEM)

**Fig. 6:**
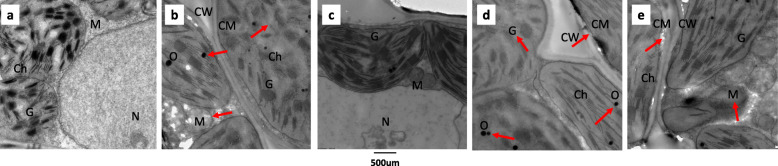
Ultrastructure of rice mesophyll cells. **a**: control group. **b**: drought stressed group. **c**: TCONS_00021861 overexpression group. **d**: miR528-3p overexpression group; **e**: TCONS_00021861 and miR528-3p co-overexpression group. Red arrows indicate the damaging effect of drought stress. Ch: chloroplast, CM: cell membrane, CW: cell wall, G: grana, M: mitochondrion, N: nucleus, O: osmiophilic granules

The ultrastructure of mesophyll cells was observed using TEM. As indicated in Fig. 6a, in control group, the morphology of mesophyll cells appeared to be normal. The chloroplasts were elliptical. The basal thylakoid sheets were neatly stacked and connected with the interstitial thylakoid, and the envelope was intact. The shape of mitochondria was regular with clear double membranes and tubular spines. The nucleus was elliptical with clear and complete nucleolus.

In drought stressed group however, many typical signs of damage were observed (Fig. 6b) especially in chloroplasts, mitochondria, including the disrupted cellular and chloroplast membranes, mitochondrial swelling, globular chloroplasts, disorder of thylakoid arrangement, and a reduced thickness of granal stacking. The nucleus is relatively stable with slight deformation. Part of the nucleus melts and becomes an empty nucleus.

Compared with drought stressed group, the cellular organelles were less affected in the TCONS_00021861 overexpression group (Fig. 6c). The shape of chloroplast appeared slightly changed and the granal stacking remained intact, suggesting alleviated cell damage after TCONS_00021861 overexpression. In the miR528-3p overexpression group (Fig. 6d), the cellular and chloroplast membranes were distorted. The grana lamellae became disintegrated and osmiophilic granules increased, which were the signs of damage to chloroplast ultrastructure. When TCONS_00021861 and miR528-3p were coexpressed, as shown in Fig. 6e, the damage effect was somewhat similar to that of the control with drought stress, including mitochondrial swelling and disrupted chloroplast membranes. Compared with miR528-3p overexpression lines however, the number of osmiophilic granules reduced and thylakoid arrangement was relatively normal, indicating that the degree of damage was relatively minor.

## Discussion

Dwindling water resource is a main challenge for the quality and quantity of rice production. lncRNAs are essential regulatory components in response to abiotic stresses. Previous studies have well-characterized lncRNAs in a set of plant species by high-throughput sequencing [3–8, 10].

Sequestration of miRNAs via target mimicry represents an important post-transcriptional regulation function of lncRNAs [22]. Several lncRNA acting as eTMs have been reported in plants under various abiotic stress [5, 17–24]. Nevertheless, most of them were merely identified by expression correlation and bioinformatic prediction. The detailed regulatory mechanisms of lncRNAs remain largely unknown.

This study provided a systematic perspective on the regulatory function of lncRNAs in the drought tolerance of *Oryza Sativa*. A lncRNA-miRNA-mRNA interaction network was comprehensively integrated via high throughput screening and bioinformatics analysis.

Using RNA sequencing technology, we identified 542 unique lncRNAs based on strict criteria, which constitute a credible group of rice lncRNAs. Among them, we identified 98 lncRNAs that were differentially expressed under drought stress.

To investigate the possible functions of the lncRNAs as ceRNAs, we constructed the potential regulatory network of lncRNA-miRNA-mRNA considering the similarity of the miRNA regulatory patterns and the similarity in expression. We next filtered the key DE mRNAs based on the literature and functional annotation. As a result, a drought-responsive ceRNA network with 40 lncRNAs, 23 miRNAs and 103 mRNAs was built. TCONS_00021861/miR528-3p/YUCCA7 triplet is located at the hub of the ceRNA network, with the highest degree and the most significant positive correlation. We then explored the comprehensive functional landscape of the TCONS_00021861/miR528-3p/YUCCA7 triplet.

There have been several reports about miR528 and members of the YUCCA (YUC) family in drought resistance. miR528 was found to be deregulated and participate in the regulation of defense response to drought stress in Sugarcane [25] and Brachy podium [26]. The YUCCA gene family, which encodes a flavin monooxygenase-like enzyme, mediates auxin overproduction that endows plants with drought resistance. For example, activation of YUCCA7 enhances drought tolerance in *Arabidopsis* [27]. Transgenic poplar plants [28] and potato [29] expressing YUCCA6 exhibits high-auxin induced phenotypes and increased tolerance to water deficit.

In line with previous findings, this study found that lncRNA TCONS_00021861 and YUCCA7 were co-expressed and dramatically down-regulated, whereas miR528-3p expression was upregulated in the PEG-treated rice compared with the controlled group. Their differential expression and coregulation were verified experimentally by RT-qPCR (Fig. 4a). Further, TCONS_00021861 showed strong positive correlation (*r* = 0.7102) with YUCCA7 and strong negative correlation with miR528-3p (*r* = − 0.7483) (Fig. 4b). RLM-5’ RACE results indicated that both TCONS_00021861 and YUCCA7 had adequate sequence complementarity with miR528-3p (Fig. 4c), and might compete binding with miR-528‐3p via shared MREs.

Credibility of the TCONS_00021861/miR528-3p/YUCCA7 regulation was further characterized in transgenic plants. TCONS_00021861 is a positive regulator of YUCCA7 *in vivo* because transcript level of YUCCA7 increased in the transgenic plant overexpressing TCONS_00021861 relative to wild type. In contrast, the overexpression of miR528-3p reduced YUCCA7 level and this reduction was rescued by simultaneous overexpression of TCONS_00021861 (Fig. 4d). These data indicate that TCONS_00021861 could regulate the expression of YUCCA7 by sponging miR528-3p.

IAA is an important plant auxin that is synthesized by YUCCA family members. IAA plays major roles in many plant growth and developmental processes, which can be synthesized via Trp-dependent or Trp-independent routes [30]. Genomic and functional studies in *Arabidopsis* have shown that *de novo* IAA synthesis was dominated by Trp-dependent pathway. Mounting evidence shows the involvement of YUCCA7 in Trp-dependent IAA synthesis. YUCCA7 catalyzes the conversion of TAM to NHT, a rate-limiting step in auxin synthesis in multiple plants. Functional assay revealed that TCONS_00021861 could induce characteristic auxin overproduction phenotypes by increasing the expression level of IAA, which could be reversed by miR528-3p overexpression (Fig. 4e). These observations signify the probable role for TCONS_00021861 in auxin biosynthesis. Consistent with this notion is the alleviated mesophyll cell damage observed by TEM in the transgenic plants overexpressing the TCONS_00021861 (Fig. 6).

A further correlation analysis was conducted between the IAA content, TCONS_00021861, miR528-3p and YUCCA7 expression level to confirm their regulatory relationship. As illustrated in Fig. 4f, there was a significant positive Pearson correlation between IAA amounts and TCONS_00021861 as well as YUCCA7 expression level, whereas miR528-3p was negatively correlated, suggesting that TCONS_00021861 mainly regulates IAA biosynthesis via decoying miR528-3p and subsequently increasing the level of YUCCA7.

**Fig. 7:**
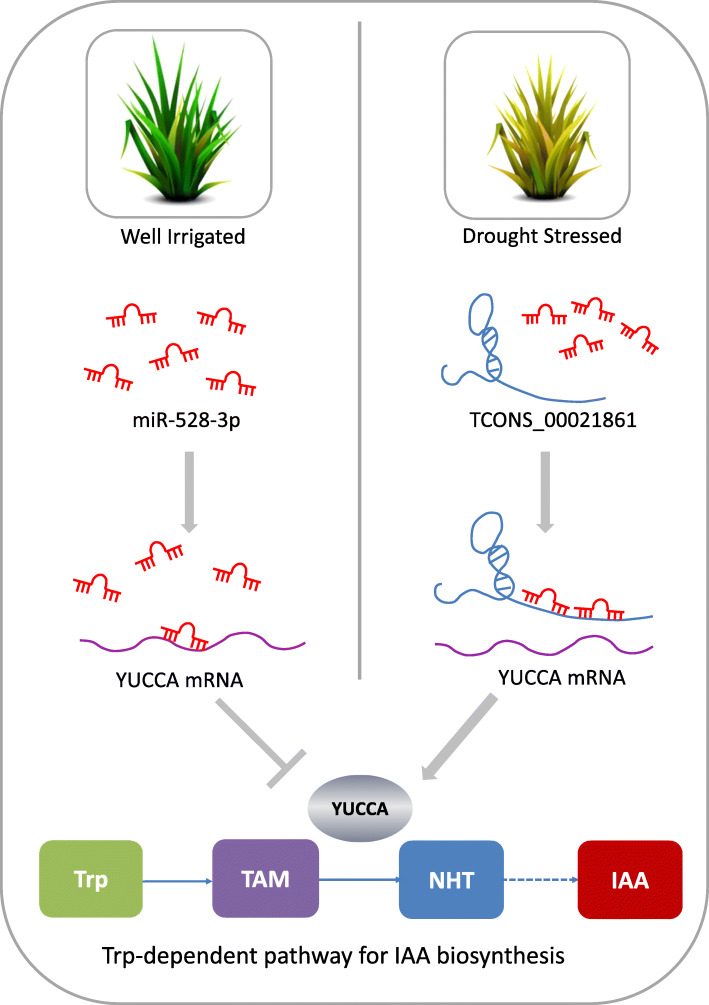
Putative model for the function of TCONS_00021861 in IAA biosynthesis under drought stress

Based on the results above, we proposed a model for the involvement of TCONS_00021861/miR528-3p/YUCCA7 in the regulation of Trp-dependent pathway for IAA synthesis (Fig. 7). In this model, drought induces the overexpression of TCONS_00021861, which acts as a specific decoy for miR528-3p and upregulates YUCCA7. YUCCA7 is involved in Trp-dependent IAA biosynthesis as a rate-limiting step converting TAM to NHT. Upregulation of YUCCA7 thus rendered a greater accumulation of IAA in rice under water deficient conditions, promoted the auxin overproduction phenotypes and ultimately endowed rice with drought resistance.

## Conclusions

In summary, this study constructed a ceRNA regulatory network for lncRNA-miRNA-mRNA interactions and found TCONS_00021861 as positive regulator of YUCCA7 gene through interaction with miR528-3p. Functional elucidation of TCONS_00021861 highlights its important roles in the regulation IAA biosynthesis in response to water deficiency, which provides a foundation for understanding of the mechanism of lncRNAs in drought response.

## Methods

### Rice culture and drought stress

The rice cultivar (*Oryza sativa* L. subsp. Japonica cv. Suxiang 3) used for this study were purchased from Zhongnongke company, Jiangsu, China. The seeds were grown in the greenhouse under 16/8 h photoperiod, 26 °C /18°C (day/night), 55 % RH, and light intensity of 250 µmol m^− 2^ s^− 1^. Six-leaf stage seedlings were exposed to 20 % PEG 6000 for 48 h to simulate drought stress of -0.5 MPa osmotic potential. The control group was cultured under well irrigation.

### Library construction and sequencing

Total RNA was extracted from rice leaves using TRIzol (Invitrogen) following the manufacturer’s protocols. For each sample, 1.5 µg RNA was used for subsequent analysis. Ribo-Zero™ Magnetic Kit was used to remove rRNAs. The strand-specific sequencing library was generated for 150 bp paired-end run on Illumina HiSeq 4000 platform, at a depth of 120 million reads per library. The stranded RNA-seq data have been submitted to the NCBI SRA with accession number SRP316304.

### Sequencing data analysis

After removing adapter contaminations or poly-N and low-quality sequences (Q20 < 20) from the raw reads, clean reads were mapped to the rice reference genome with Tophat2 [31] and assembled using Cufflinks (v2.1.1) [32] to construct transcriptomes. CPAT [33], CNCI [34] and CPC [35] and were used to filter transcripts with coding potential. Coding transcripts with CPAT=“yes”, CNCI scores > 0 and CNCI scores > 0 were removed.

Expression levels of lncRNA and mRNA were determined with RPKM, and miRNA expression level was calculated with transcript per million (TPM). The differential expresion between groups was determined by DEGseq (adjusted *P*-value < 0.05 and log_2_FC > 1).

### Construction of the ceRNA network

Nucleotide sequence-based miRNA-target interaction was predicted by miRanda tools with default parameters. Coexpression-based miRNA‐target interaction was determined by calculating Pearson correlation coefficient (PCC) for miRNA-target pairs. Negatively correlated pairs PCC<-0.7 and *p* < 0.01were selected for further analysis. Overlapping results of miRanda and coexpression thus represent a candidate miRNA‐target pair.

Based on the ceRNA principle, we calculate the ceRNA score of an lncRNA-mRNA pair targeted by common miRNAs according to the following formula:

$$\text{c}\text{e}\text{R}\text{N}\text{A} \text{s}\text{c}\text{o}\text{r}\text{e}=\frac{\text{M}\text{R}\text{E} \text{f}\text{o}\text{r} \text{s}\text{h}\text{a}\text{r}\text{e}\text{d} \text{m}\text{R}\text{N}\text{A}\_\text{l}\text{n}\text{c}\text{R}\text{N}\text{A}}{\text{M}\text{R}\text{E} \text{f}\text{o}\text{r} \text{l}\text{n}\text{c}\text{R}\text{N}\text{A}}$$

The lncRNA-mRNAs correlation was analyzed using the PCC from matched lncRNA and mRNA expression profiles. The lncRNA-mRNA pairs with PCC > 0.7 and *p* < 0.01 were considered to have potential relevance. The shared pairs from ceRNA scoring and coexpression were considered as the true ceRNAs, and were then mapped using Cytoscape software (V.3.5.1) [36] to visualize the lncRNA-miRNA‐mRNA network.

### qRT-PCR

miRNA, lncRNA and mRNA expression levels were analyzed by qRT-PCR. The actin gene was used as an internal control and the 2 − ΔΔCt method was used to quantify the expression level. All PCR reactions were performed by three technical replicates and three biological replicates.

### RNA Ligase-Mediated 5’ RACE(RLM-5’ RACE)

RLM-5’ RACE was performed using 5’-Full RACE Kit (Takara, Japan). Briefly, 5 µg total RNA was ligated to RNA adapter followed by reverse transcription using the Oligo (dT) primer and superscript reverse transcriptase. PCR was performed with 3’ gene-specific primers and 5’ adaptor primers. The PCR product was cloned into pEASY-T1 vector and plasmid DNA was sequenced.

### Vector construction and plant transformation

PCR product of miR528-3p was cloned into the KpnI and PstI sites of plant expression vector p1301-35 S-NOS. The full-length sequences of lncRNA were amplified and inserted into the pEASY-Blunt vector. After Sanger sequencing, the correct fragments were digested with XhoI and KpnI and cloned into the PMV2 vector. These plasmids were transformed into rice plants by the floral dip method [37]. The whole pMV2 plasmid was transfected as a negative control. T0 transgenic plants were grown to maturity. T1 seeds were harvested and germinated on half MS medium containing 25 mg/L hygromycin. T1 progenies were analyzed by PCR for the segregation patterns using TCONS_00021861 and miR528-3p specific primers. T2 seeds from independent T1 lines showing 3:1 Mendelian segregation ratios were harvested separately and were germinated on hygromycin-containing rooting medium. T2 seedlings with absence segregation patterns were considered to be homozygous lines and were then screened and confirmed by RT-PCR analysis. Among the positive T2 transformants screened, the homozygous lines with highest expression level of the target transgene (named as LNC_5-1-4,_ MIR_9-1-2,_ LNC + MIR_4-1-1_) were used for subsequent functional analyses.

### Measurement of growth indices, ROS, IAA and TEM

Growth was evaluated in terms of elongation and quantity of leaf and root 48 h after stress. The H_2_O_2_ and O_2_•^−^ content were determined following previous research methods [38]. For IAA content quantification, 5 g leaves was homogenized and extracted with methanol and ethyl acetate. Extract of leaves was then subjected to HPLC on Agilent Zorbax Eclipse XDB-C18 column (250 × 4.6 mm, 5 μm). TEM images of mesophyll cells were obtained (FEI Tecnai Spirit) according to Ju et al. [39].

### Statistical analysis

All data were analyzed using SPSS v22.0. Student’s t-test was performed to compare significant differences between the two groups of data. Data were presented as SD calculated from three replicates.

## Supplementary Information

**Additional file 1.** Statistics of raw, clean and mapped reads of all 6 samples.

**Additional file 2.** Full list of lncRNA-miRNA-mRNA interactions in drought responsive ceRNA network.

**Additional file 3.** Oligonucleotide primers used in RT-qPCR.

**Additional file 4.** The specific primer sequences for RLM-5’ RACE of target genes.

**Additional file 5.** Genetic analysis of the T1 progeny of transgenic rice lines.

## Data Availability

The RNA-seq data have been submitted to the NCBI SRA database with accession number SRP316304 (https://trace.ncbi.nlm.nih.gov/Traces/sra/?study=SRP316304). Other datasets supporting the conclusions of this article are included within the article and its additional files.

## Abbreviations

ceRNA: Competing endogenous RNA
eTMs: endogenous miRNA target mimics
IAA: Indole-3-acetic acid
lncRNA: Long non coding RNA
MRE: microRNA response element
RLM 5′RACE: RNA ligase-mediated 5′ rapid amplification of cDNA ends
